# Bridging the Gap in FDA Approval for Pediatric Neuromodulation Devices

**DOI:** 10.3390/children12020148

**Published:** 2025-01-27

**Authors:** Ammar Shaikhouni, Cameron Brandon, Cory Criss

**Affiliations:** 1 Division of Pediatric Neurosurgery, Nationwide Children’s Hospital, 700 Children’s Dr., Columbus, OH 43205, USA; 2 Department of Neurological Surgery, The Ohio State University Medical Center, Columbus, OH 43210, USA; cameron.brandon@osumc.edu; 3 Section of Pediatric Surgery, Department of Surgery, Nationwide Children’s Hospital, 700 Children’s Dr., Columbus, OH 43205, USA; cory.criss@nationwidechildrens.org

**Keywords:** pediatric neuromodulation, disparities, medical device regulation, off-label use

## Abstract

While neuromodulation devices for managing neurological conditions have significantly advanced, there remains a substantial gap in FDA-approved devices specifically designed for pediatric patients. Devices like deep brain stimulators (DBS), vagus nerve stimulators (VNS), and spinal cord stimulators (SCS) are primarily approved for adults, with few options for children. To meet pediatric needs, off-label use is common; however, unique challenges to pediatric device development—such as ethical concerns, small trial populations, and financial disincentives due to the limited market size—continue to hinder progress. This review examines these barriers to pediatric neuromodulation device development and FDA (Food and Drug Administration) approval, as well as the current efforts, such as FDA initiatives and consortia support, that address regulatory and financial challenges. Furthermore, we discuss pathways like the Humanitarian Device Exemptions and Real-World Evidence programs that aim to streamline the approval process and address unmet clinical needs in pediatric care. Addressing these barriers could expand access to effective neuromodulation treatments and improve patient care.

## 1. Introduction

Neuromodulation devices are devices that interact with neural tissue—either peripherally or centrally—through electromagnetic or pharmacological means to alter nerve activity for therapeutic purposes. They include devices that deliver pharmacological or electrical stimuli in response to recorded neural or other signals or ones that alter neural activity in an open-loop fashion [1]. 

Efforts to modulate nerve activity using electrical or chemical means date back centuries, but practical implantable devices only emerged in the 1990s with the development of deep brain stimulation (DBS) for treating essential tremor [1]. Commensurate with improvements in computer technology and materials, the last two decades have seen a fast increase in the development of neuromodulation devices [1]. These various devices have demonstrated efficacy in the adult population and indications for neuromodulation continue to expand [1]. 

Although many pediatric conditions have shown responses to neuromodulation in adults, device use among pediatric patients remains limited [2]. Conditions such as intractable epilepsy, psychiatric disorders, movement disorders, and chronic pain affect both pediatric and adult patients, posing significant burdens on the healthcare system as well as on the families and caregivers of affected children. These conditions are frequently resistant to medical therapy [2,3,4]. Treatment with neuromodulation devices may fill this gap and have the potential to have a large impact on the lives of these children, their families, and the medical system. 

This review examines the gaps in neuromodulation device use between pediatric and adult patients, identifies barriers to pediatric device development and approval, and discusses efforts aimed at bridging this gap to advance neuromodulation treatment options for children.

## 2. FDA-Approved Neuromodulation Devices

### 2.1. Neuromodulation Devices for Adult Use Only

Advances in neuromodulation have opened new avenues for managing many neurological conditions. Unlike traditional pharmacological approaches, neuromodulation provides targeted and adaptive interventions that can modulate central and/or peripheral neural activity directly. These techniques offer promising alternatives for patients with neurological disorders not responsive to pharmacological interventions and in some cases avoid the need for more invasive ablation or tissue resection surgeries. Table 1 lists current FDA-approved neuromodulation devices. In this and the following two sections, we discuss the current neuromodulation devices approved by the United States Food and Drug Administration (FDA) and briefly discuss their development history, clinical applications, and efficacy. We will focus on invasive neuromodulation devices that use electricity to modulate the central or peripheral nervous system activity to treat neurological or psychiatric diseases. We will not cover devices that use neuromodulation to treat respiratory function, such as phrenic nerve stimulation or hypoglossal nerve stimulators. We will not cover noninvasive neuromodulation devices, such as transcranial magnetic stimulation (TMS), and devices that use pharmacological agents to alter neuronal activity, such as intrathecal pumps. Devices that are used to restore sensory function with electrical stimulation such as cochlear implants are also not discussed here.

One category of FDA-approved neuromodulation devices alters neural activity by stimulating neurons using electrodes implanted into targeted brain regions containing these neurons’ cell bodies or their axons [5]. Those devices include deep brain stimulation (DBS) and responsive neurostimulation (RNS) devices [5,6]. A DBS system consists of one or two leads consisting of four or more contacts each that are connected to a pulse generator typically implanted in the chest wall. An RNS system consists of two leads each with four contacts connected to a pulse generator implanted in the skull. In both systems, electrical stimulation is delivered to disrupt neural activity in the implanted neural tissue or its connected neural network [7]. The RNS devices are closed-loop systems, while DBS are currently used as open-loop devices. In RNS, the device is capable of recording neural activity, and the electrical stimulation is applied in response to detected seizures rather than through the open-loop continuous stimulation strategy currently used in DBS systems. In 1997, Medtronic became the first company to receive FDA approval for a DBS device targeting the thalamus to treat essential tremor and tremors associated with Parkinson’s disease (PD). This was followed by the 2002 FDA approval of DBS targeting bilateral stimulation of the subthalamic nucleus or globus pallidus pars interna (GPi) for the treatment of symptoms associated with PD [5,8,9,10]. The approval was based on multiple studies that showed that DBS provides better motor function control and improved quality of life when compared to the best medical therapy in patients diagnosed with PD [8,9,11] or essential tremor [9,12]. In 2009, DBS of the anterior limb of the internal capsule was also approved under an HDE for the treatment of obsessive-compulsive disorders (OCD) based on accumulating evidence showing possible efficacy with low risk from DBS [13,14,15,16]. This marked the first approval of a DBS device outside movement disorders. Exploration of DBS of the thalamus to treat generalized and focal epilepsy dates back to the 1980s [5,17,18,19,20,21,22,23]. However, the Neuropace RNS device, approved in 2013, was the first approved neuromodulation device for the treatment of drug-resistant epilepsy [24,25]. This system continuously records brain activity and uses proprietary algorithms to detect and respond to seizures using brief electrical stimulation that halts seizure evolution. In 2018, the FDA approved the use of DBS of the anterior nucleus of the thalamus (ANT) for the treatment of DRE after the publication of the SANTE trial’s long-term results showing efficacy and low side effects [26,27]. Additional DBS devices from Boston Scientific and Abbott Laboratories are now FDA-approved. This has led to rapid increases in the improvement of DBS pulse generators and electrode technologies that last longer, allow more selective electrical stimulation of neurons, and respond to changes in brain electrical activity like RNS [5]. As a result of this, there have been more than 160,000 DBS devices implanted worldwide [5]. Many trials are in the process of exploring the use of new brain targets for DBS or RNS for the treatment of DRE, movement disorders, and psychiatric diseases. Except for DBS in the treatment of primary dystonia, discussed below, those devices are FDA-approved for use in adult patients only. 

The FDA also approved neuromodulation devices to deliver stimulation for the treatment of chronic pain that has not responded to multimodal pharmacological and psychiatric treatments. These devices include spinal cord stimulation (SCS) and peripheral nerve stimulation (PNS) devices. Both devices were developed prior to the FDA gaining regulatory authority for medical devices in 1976 [28,29,30]. SCS involves implanting electrodes in the epidural space to stimulate the dorsal columns of the spinal cord. This stimulation is thought to inhibit the transmission of pain signals to the brain by disrupting sensory processing circuits in the spinal cord [28,31]. SCS has been shown to be effective in the treatment of back and leg pain [28]. In contrast to SNS electrodes, PNS electrodes are placed near peripheral nerves associated with pain, delivering electrical impulses to disrupt pain signals closer to the source. PNS is, therefore, particularly useful for pain that is localized or specific to certain body regions, such as nerve injuries, and has shown effectiveness in conditions like migraines and post-amputation pain [28,32,33]. The FDA approved SCS for use in adults in 1984. PNS systems were first FDA-approved for adults in 1989. Both techniques have advanced significantly in recent years, particularly with the advent of percutaneous implantation methods and ultrasound guidance, making them less invasive and more accessible [28]. Together, these neuromodulation techniques provide clinicians with versatile tools for managing pain in adult patients who previously had limited treatment options. 

Neuromodulation devices have also found use in the treatment of bowel and bladder disorders. These currently include sacral nerve stimulation (SNS), approved by the FDA in 1997 for the treatment of overactive bladder and, later, urinary and fecal incontinence. SNS devices use electrodes placed near sacral nerve roots and connected to an implanted pulse generator. This is performed to improve control of the pelvic floor by the modulation of efferent nerves. However, the full mechanism of action is not clear, and some investigators suggest that the mechanism of action is through stimulation disruption of central afferent signals [34]. SNS has been found to be an effective and safe treatment for urinary urge incontinence and fecal incontinence [34]. Another implantable neuromodulation device for the treatment of urinary incontinence was FDA-approved in 2022. The eCoin (Valencia Technologies Corporation, Valencia, CA, USA) implanted tibial nerve stimulator (iTNS) is a nickel-sized device that includes a power source and a signal generator implanted near the tibial nerve in the lower leg. It uses electrical stimulation applied to the tibial nerve to affect the bladder function indirectly. Electrical pulses travel up the leg along the tibial nerve to reach the sacral plexus, where it is thought to disrupt the overactive signals driving bladder incontinence [35]. Clinical data has shown that it can match SNS devices in clinical efficacy with less invasive implantation and potentially fewer side effects, but long-term results are not yet clear. 

### 2.2. Neuromodulation Devices for Both Adult and Pediatric Use

In contrast to the large number of neuromodulation devices approved by the FDA for the treatment of adults, there are fewer neuromodulation devices approved by the FDA for use in adults and pediatric patients. These include cochlear implants, vagus nerve stimulators (VNS), and DBS devices designed for the treatment of primary dystonia. 

In addition to movement disorders, the FDA approved DBS for use in the treatment of patients with primary dystonia under humanitarian device exemption (HDE) in 2002. The HDE process was devised by the FDA to allow the approval of medical devices that are deemed to be safe for use but may not have strong data for efficacy due to the difficulty in conducting clinical trials on the use of the device. Under this mechanism, DBS was approved for the treatment of patients with primary dystonia aged seven years and older. The decision to approve the device was supported by data showing that DBS of the bilateral GPi is effective in reducing motor symptoms in patients with primary dystonia, including associated improvement in general health and well-being with relatively low risks [36,37,38,39]. Dystonia remains the only FDA-approved disease indication for DBS use in pediatric patients. 

For pediatric patients, vagus nerve stimulation (VNS) is the only FDA-approved neuromodulation technology for the treatment of DRE. VNS is a device that consists of one lead connected to a small pulse generator implanted in the chest. The lead has two electrical contacts that are wrapped around the left vagus nerve in the neck. The mechanism of action of VNS is not well understood. It is thought that stimulation of the afferent pathways within the vagus nerve is transmitted to deep brain nuclei, where it modulates the activity of widely projecting neurons, leading to desynchronization of epileptiform activity and reduction of seizure severity and/or frequency [40,41,42,43]. VNS was approved for use in patients 12 years of age or older by the FDA in 1997 based on trial results that showed that an estimated 30–40% of patients with VNS implants achieve a 50% or greater reduction in seizures with low adverse side effects [44]. Similar success was later demonstrated in the pediatric population in one randomized trial and multiple observational studies [45,46,47,48,49]. Relying on premarket approval data from multiple clinical trials that included patients younger than age twelve as well as post-approval surveillance data, the FDA approved the use of VNS for the treatment of DRE for patients aged four and older in 2017. Because RNS and DBS are FDA-approved neuromodulation treatment options for adults only, this leaves pediatric patients with VNS as the only on-label option for the treatment of DRE. For patients under four years of age, there are no FDA-approved neuromodulation devices available for DRE treatment.

### 2.3. Off-Label Use of Neuromodulation Devices in Pediatrics 

As seen above, there is a gap between pediatric and adult neuromodulation device approval. Surveys of specialists treating rare diseases showed that 74% of the device needs are related to pediatric devices [50]. This results in the disproportionate use of many medical devices that are authorized for adults being used in children or “off-label”. This improvisation has become the most common method for neuromodulation devices to be used in pediatrics. This practice remains legal, supported by the American Academy of Pediatrics, and the FDA considers it a part of medical practice, thus not regulating it [51]. Here, we describe the most common off-label uses of neuromodulation devices in pediatrics. 

Pediatric movement disorders are rare, but many have phenotypes that overlap with those seen in adult patients. Many are treated pharmacologically in the same way in adult and pediatric patients. When these symptoms become resistant to medical therapy, many physicians have used DBS devices to help in treatment. The use of DBS in pediatric patients is on-label for patients over the age of seven and with primary dystonia, but DBS is also used off-label with varying efficacy [3,52,53,54,55,56,57]. In some cases, the off-label use is based on lessons learned from similar DBS off-label use in adults that was met with some success, such as in Tourette’s syndrome [58,59,60,61,62]. In other cases, the treatment was used without evidence from adult cases, such as in various cases of pediatric-onset movement disorders [63]. 

For epilepsy, the use of DBS and RNS devices for the treatment of DRE in pediatric patients is now a common practice [64,65]. In addition, many epilepsy centers are using these devices to treat generalized epilepsy cases [36,66,67,68,69,70] and often target deep thalamic structures other than the ANT (REF). VNS devices are also used in children under the age of four, with reported success rates like those seen in on-label use in patients aged four and over [71]. Because of the increasing off-label use of these devices in the treatment of DRE, three clinical trials are investigating the use of the RNS device in generalized epilepsy, Lennox-Gastaut syndrome, and focal epilepsy in patients aged twelve or older [72]. If these trials prove efficacious, this may result in FDA approval of the use of these devices in pediatrics for more indications. 

For the treatment of pain, both spinal cord stimulation and peripheral nerve stimulation have been used in adolescent patients. Off-label use of SCS in pediatric patients was reported to decrease pain intensity, reduce medication use, and improve functional outcomes in a variety of chronic neuropathic pain etiologies [73,74], but most reports are single case studies or small retrospective reviews with risk of bias. Even fewer reports exist on the use of PNS for the treatment of pain in pediatric patients [75]. Almost all reported case reports are in patients over the age of ten. This is likely due to the difficulty in assessing pain in younger patients who may also have concomitant developmental delays. 

Neuromodulation devices are also used off-label to treat urological disorders in kids. SNS has been shown to improve bladder function in children with refractory voiding issues that do not respond to standard medical treatments. One study involving 26 children (average age ~10.8 years) found significant improvements in urodynamic parameters with the use of SNS [76]. In another study, SNS significantly reduced fecal and urinary symptoms in 29 children and allowed some to discontinue medications or reduce other interventions [77]. These results were shown to be durable on long-term follow-up and safe but with complications like those seen in adult patients [78,79]. 

The above shows that off-label use of neuromodulation devices has provided crucial treatment options for pediatric patients, showing promise in managing severe conditions. However, the limited availability of on-label devices tailored for children underscores the need for dedicated research and development as the growing use of off-label devices creates an issue where both the perceived risk and benefit to the child are unclear.

## 3. Barriers to Developing Pediatric Neuromodulation Devices 

Advancing the development of on-label pediatric neuromodulation devices could offer safer and more effective treatments and help eliminate unsafe and cost-ineffective alternatives. However, significant barriers hinder the development of pediatric neuromodulation devices.

### 3.1. Regulatory Challenges

Medical devices are regulated for intended use and level of risk by the Center for Devices and Radiological Health (CDRH) of the FDA. The CDRH classifies medical devices into three classes in ascending order of the risk they pose to patients [80,81]. Class III devices support or sustain human life, are implanted, or present a potential and unreasonable risk of illness or injury. This class includes most neuromodulation devices and requires the most intensive regulatory process prior to approval.

FDA approval of class III devices requires a scientific and regulatory review called premarket approval (PMA). Premarket approval is an involved process that provides scientific evidence to support the claim that a new device provides health benefits that outweigh the associated risks. This usually takes the form of at least one clinical trial, often referred to as the “pivotal” trial [82]. These trials must be designed in compliance with the standards of Good Clinical Practice [83], ensuring that ethical and scientific standards are met with a focus on the protection of research subjects before testing the device with human subjects. 

Because children and adolescents are vulnerable populations, clinical trials to develop neuromodulation devices targeting them must address the ethical, epidemiological, physiological, and developmental issues specific to the pediatric population, as well as the indirect financial challenges that addressing these issues creates.

### 3.2. Infrastructure Challenges in Pediatric Clinical Trials

One major barrier to the development of pediatric neuromodulation devices is the lack of sufficient clinical trial infrastructure tailored to pediatric needs. Many institutions do not have specialized resources for pediatric device trials, such as institutional review boards (IRBs) experienced in pediatric device protocols, study coordinators, and data management systems customized for younger populations. Additionally, recruiting specialized staff with expertise in pediatric device trials is challenging, making it difficult to conduct the large, multi-institutional studies often required for FDA approval. This infrastructure gap contributes to higher costs, complex logistics, and delays in trial timelines, ultimately slowing down access to neuromodulation therapies for pediatric patients.

### 3.3. Ethical Barriers

All clinical trials designed to study pediatric neuromodulation devices need to address informed consent. Pediatric patients do not have legal standing to make medical decisions. Their parents must consent to the treatment legally, but they may be uncomfortable doing so if the device benefits or risks are not well understood or characterized. The pediatric patient must also assent to the implantation of the neuromodulation device. This is important, as clinical trials require the cooperation of the patient with many visits to report positive and negative effects. This is especially true for patients suffering from neurological diseases that may have further developmental or cognitive concerns that further limit their ability to assent or participate effectively. 

Consent and assent are challenging to obtain even in cases of proven neurosurgical procedures and, therefore, will be more challenging for novel devices with unknown efficacy or side effects [4,84]. There are recommended strategies to address this challenge, which are really an individualized and targeted approach to each situation. While pediatric patients do not have a specific legal capacity to make decisions, it is important to attempt assent and individualized communication efforts in these circumstances. This also means that investigators designing trials to test the efficacy and risk of pediatric neuromodulation devices need to explain the trial in age-appropriate language for pediatric patients and develop strategies to help make the trial follow-up visits and reporting of outcomes and adverse events appropriate for pediatric patients. This is crucial to secure enrollment of enough subjects and to avoid an expensive trial failure due to a lack of recruitment. 

### 3.4. Epidemiological Barriers 

Ensuring a high recruitment rate for trials is always desirable but it is especially important for trials of pediatric neuromodulation devices due to the low number of potential pediatric candidates for such devices. Children under 18 years old constitute about 22% of the U.S. population, totaling approximately 73 million individuals [85]. However, the vast majority are generally healthy and do not require specialized medical devices like neuromodulation implants. Neurological conditions necessitating such interventions, such as epilepsy or movement disorders, are relatively rare in children. For example, about 470,000 children in the United States have active epilepsy, representing roughly 0.6% of the pediatric population [86]. This limited number of patients makes clinical trials both expensive and logistically complex. To obtain sufficient enrollment, trials often need to be conducted across multiple centers, increasing costs and extending timelines for the trial sponsors. 

### 3.5. Clinical and Technical Barriers 

The pediatric patient population is continuously changing as children grow and develop. The nervous system also undergoes significant changes into early adulthood [87]. This means that pediatric neuromodulation device trials need to not only consider issues related to the child’s physical growth but also need to address how neuromodulation devices interact with the developing nervous system, which is unique to this patient population. 

Pediatric neuromodulation devices need to address the effects of physical growth on the performance of the device. For example, the growth of a child’s brain may result in moving an intracranial electrode away from its stimulation target. Electrodes implanted in the peripheral nervous system or for epidural spinal cord stimulation may get stretched with body growth. These electrodes may fracture or move away from the target structure, as is commonly seen with ventriculoperitoneal shunts [88]. Because this will affect stimulation efficacy, neuromodulation devices may need adjustments or replacements to accommodate growth. Another challenge is the use of a single-device design for both adult and pediatric patients. Many of the currently approved neuromodulation devices use a one-size-fits-all design strategy to reduce regulatory and manufacturing costs. The use of adult-sized devices in children is known to have increased side effects. The most common side effects seen with this approach are skin breakdown and infection at a higher rate than in the adult population [4,89]. This complicates the long-term viability and “future-proofing” of neuromodulation devices.

Additionally, the pediatric nervous system has significantly greater potential for neuroplasticity as compared to the adult brain. The human brain undergoes its greatest changes in size and connectivity during the first two decades of life [87]. As children grow, some of their conditions may improve or worsen with age. For example, some psychiatric diseases such as OCD that may be targeted by DBS in adults might not be good indications in pediatric patients [90]. We also do not have a good understanding of the development of the pediatric nervous system or the effects of stimulation and plasticity on its structure. Without sufficient clinical trials focused on the pediatric population, it is difficult to understand the long-term safety of neuromodulation in the developing brain. This is partly due to the lower pediatric research funding compared to funding for adult research [91,92]. Whether this proves to be deleterious or advantageous in the treatment of various conditions remains to be seen [2,4]. In some instances, such as brainstem cochlear implants, it is thought that implanting the device early harnesses the powerful plasticity of a young brain and provides better long-term outcomes [93]. Following this logic, if a device is implanted early in a child’s development and is effective in improving that child’s movement, communication, or interaction with the world, one would expect improved cognitive and social development. This benefit of early intervention remains largely theoretical, and one may find that continued stimulation over decades of life may lead to decreased efficacy through habituation or may result in unintended side effects due to the nervous system’s plasticity. What is certain is that there is increased uncertainty in the outcomes of trials of pediatric neuromodulation devices due to the unknown effect of stimulation in the developing brain. 

### 3.6. Financial Barriers

The above barriers make the development of medical devices expensive and lengthy. However, they also limit the market size of pediatric neuromodulation devices. The small market size reduces financial incentives for companies to invest in developing pediatric neuromodulation devices. The small market size is also distributed over a large geographic area. This exposes manufacturers to uncertainty in device reimbursements due to the heterogeneous device coverage decisions that vary from state to state, in addition to higher sales and support costs [81,94,95]. In addition, pediatric medical care is traditionally reimbursed at a lower level than in adults across the board, putting further downward pressure on potential revenues [96]. Therefore, the high research and development costs may not be recouped through sales in such a limited and complex market, making it less attractive for manufacturers to pursue approval and labeling for pediatric neuromodulation devices. This relatively low return on investment for pediatric neuromodulation devices compared to adult neuromodulation devices is often cited as the dominant barrier to pediatric neuromodulation device development [97].

## 4. Bridging the Gap: Efforts to Advance Pediatric Neuromodulation Device Development

These various challenges in pediatric devices have resulted in a significant and measurable deficit in the approval of pediatric devices. Pediatric device approval lags 5–10 years behind the adult market and only a small fraction of all approved devices are approved for pediatric use [82,98]. Among these, most are approved for older children and adolescents and rarely for neonates and infants [95]. As such, there has been national recognition of the barriers to pediatric device development and certain measures have been taken to provide alternative pathways to bring neuromodulation devices to meet the clinical needs of pediatric patients.

### 4.1. FDA Programs Supporting Pediatric Device Development

The Food and Drug Administration Amendments Act of 2007 contained incentives to facilitate the development of medical devices for pediatric populations (defined as patients who are younger than 22 years of age). One of the programs implemented to address the challenges of pediatric patients with smaller patient populations is the Humanitarian Use Device (HUD) designation. The reason for this specific designation is to encourage innovation, address unmet needs, support research for rare diseases, and expedite and streamline the approval process. A device meets HUD criteria if its indication affects less than 8000 individuals per year. This clearly helps pediatric patients more than adults because of the rarity of children with severe diseases that can be treated by neuromodulation. Once a device is considered a HUD, the manufacturer can apply for Humanitarian Device Exemption (HDE). One of the main advantages of this designation includes the adjusted requirements for approval, essentially relying mainly on the safety of the device and a probable benefit. This is in comparison to the PMA process, which generally requires additional effectiveness data that are difficult to obtain in rare disease cases.

To further aid financial concerns, typical fees for the premarket approval process are waived for pediatric-specific devices (prohibited in adults). Furthermore, the HUD/HDE process also allows the manufacturer to sell the device for profit if the device has a disease indication in pediatric patients and is labeled for use in pediatric patients [99]. This only applies until an FDA-determined number of devices, known as the Annual Distribution Number, is sold for profit. After this, the manufacturer can not draw profit from the sale of additional units. Despite these changes, a review of approved pediatric medical devices reveals that they are more likely to enter clinical use without prior randomized trials and often only being tested in patients 18 years of age or older [82,95,100].

To further encourage the development of pediatric devices. The FDA enacted other programs that are focused on aiding the advancement of pediatric devices. One of these is the Breakthrough Device Program [101]. The Breakthrough Devices Program can be utilized for pediatric neuromodulation devices if they meet the eligibility criteria, which include providing a more effective treatment or diagnosis of life-threatening or irreversibly debilitating conditions. For example, pediatric devices intended for serious conditions like refractory epilepsy or severe neurological disorders may qualify, especially if they offer significant improvements over existing therapies. Participation in the program offers benefits such as priority review, interactive communication with the FDA, and assistance with efficient clinical study design, potentially reducing development costs and accelerating time to market, thereby helping to overcome financial barriers and making investment in pediatric devices more attractive despite the smaller market size and reimbursement challenges. 

Another newer pilot program is called the Total Product Life Cycle (TPLC) Advisory Program [102]. This program was launched to foster innovation and expedite patient access to safe and effective medical devices, and it offers manufacturers earlier and more frequent interactions with FDA experts from various disciplines. This collaborative approach aims to proactively identify and address potential scientific, regulatory, and policy issues at all stages, from device conception and design through clinical trials, premarket review, and post-market surveillance. Some of the resources are advice on payer coverage, physician feedback, MedTech industry experience, and regulatory expertise. By streamlining the regulatory pathway and enhancing communication, the TPLC Advisory Program aims to reduce development times and costs, ultimately facilitating the introduction of innovative medical devices, including pediatric neuromodulation devices, to the market. It is currently piloting both adult and pediatric devices and does have the requirement that they have the breakthrough designation. 

There are additional adjuncts to clinical trials specifically designed to address challenges often faced in pediatric clinical trials, such as high costs and low recruitment numbers. One of these is the Early Feasibility Studies (EFS) Program [103]. This program allows for small-scale clinical studies that assess the initial safety and functionality of a device in a limited patient population, typically when device design is not final. The program provides a more flexible regulatory framework, enabling iterative changes to the device or study protocols based on emerging data. By doing so, the EFS Program aims to accelerate the development of innovative medical technologies, particularly those addressing unmet medical needs or representing breakthrough advancements, while maintaining patient safety and promoting robust data collection.

### 4.2. The Pediatric Device Consortium: Collaborative Support for Development

One of the most widely utilized FDA initiatives is the Pediatric Device Consortia (PDC) Program [104]. This is an FDA-funded initiative established in 2007 by Congress to support non-profit consortia that promote and support pediatric medical device development along the product life cycle. These resources include and are not limited to business development, regulatory, intellectual property, translational studies, capital investment, engineering/prototyping, clinical trial planning, and reimbursement strategies. These programs have supported over 1000 pediatric device-related projects since their inception and continue to improve their efforts [105]. The most recent cycle in 2022 funded five new consortia recipients, including the Southwest National Pediatric Device Consortium (SWPDC), the Consortium for Technology and Innovation in Pediatrics (CTIP), the Alliance for Pediatric Device Innovation, the UCSF-Stanford Pediatric Device Consortium, and the Midwest Pediatric Device Consortium (MPDC) [104]. This funding cycle was focused on new initiatives, which include encouraging combined direct device awards, building clinical trial infrastructure, a commitment to under-served populations, and real-world evidence generation. All consortia give out individual direct device funding awards of up to $50,000 per project per year. This can be combined with other consortia for a maximum of $250,000 per project per year. Some of the other resources that the PDC programs provide are advocacy efforts and regulatory meetings with the FDA.

### 4.3. Increased Funding Opportunities

The innovation process remains a long and rigorous path that requires continued funding along the way. There are many funding opportunities within the public–private partnerships (PPP) available for device companies and investigators. A PPP is where the government and the private sector jointly invest in a provision of public service, which can be beneficial to both entities. This remains a vital component of medical device advancement due to the immense challenges of moving products to the market [106]. These resources can offset product advancement costs and provide funding opportunities, like the pediatric device consortium grants [107].

Aside from PPP, there are other sources for non-dilutive funding, which are opportunities that do not require companies to give up equity or ownership stakes in exchange for funds: Small Business Innovation Research (SBIR) and Small Business Technology Transfer (STTR) program grants. SBIR grants are awarded through a competitive process to small businesses with the intention to stimulate technological innovation and encourage participation by socially and economically disadvantaged businesses. STTR grants are awarded to small businesses to facilitate the transfer of innovative technologies from research institutions (such as universities and federal laboratories) to the marketplace. The application and award process for these grants can be very long and rigorous, but the grants provide a more achievable funding mechanism that has a better return rate than typical NIH funding [108]. These grants are offered through agencies such as the National Institute of Health (NIH), the National Institute of Children Health and Human Development (NICHD), the Department of Defense (DoD), and the Nationwide Science Foundation (NSF). They are structured into three phases, focusing on proof of concept, technological development, and commercialization. 

Private funding opportunities for neuromodulation devices are comparatively limited. Potential sources include non-profit foundations and organizations with missions that serve a pediatric population that may benefit from the development of the neuromodulation device. Those are usually unable to provide sufficient funds to offset the cost of development of the devices but may help if combined with other resources. While venture capital has been used to fund innovative neuromodulation devices, it has only done so for devices targeted for adult use [4]. This is not surprising given the relatively low ROI of pediatric neuromodulation devices. 

### 4.4. Initiatives to Strengthen Clinical Trials Infrastructure

Addressing the infrastructure gap in pediatric device trials is critical for advancing neuromodulation treatments. Initiatives such as the System of Hospitals for Innovation and Pediatrics-Medical Devices (SHIP-MD) aim to create a collaborative network of hospitals, industry partners, regulatory bodies, and academic institutions to support pediatric medical device development, particularly in areas like neuromodulation devices [109,110]. This complements the work by the FDA, PDC, and other PPPs who are also actively supporting infrastructure improvements by funding centers with expertise in pediatric trials, which facilitates more efficient study design and management. These efforts are helping to create a sustainable framework for pediatric neuromodulation research and development.

It is also important to highlight the extremely important exercise of utilizing clinician and provider-directed feedback and input during development and evaluation. This remains challenging for all inventors and companies as direct access to clinician feedback remains challenging. Focusing on customer discovery and end-users can be utilized in specific programs such as NSF I-Core and the pediatric device consortiums. 

### 4.5. Data Registries and Real-World Evidence in Pediatric Neuromodulation

Off-label use of neuromodulation devices is the most common way that neuromodulation devices find their way into pediatric patients. While this method is less desirable than obtaining full FDA approval, it offers a method by which data on the use of the device in pediatric patients are obtained. Data outside of traditional clinical trials and controlled settings, or real-world Evidence, can provide evidence for regulatory submissions, post-market surveillance, market evaluation, and simulated clinical trials [111]. There is increasing emphasis on this mechanism of data acquisition, especially in the pediatric population [112,113]. There are many data registries being utilized for this, including the Pediatric Epilepsy Research Collaborative (PERC), PEDSnet, Pediatric Health Information System (PHIS), The pediatric international deep brain stimulation registry project (PEDiDBS) [114], and many more.

Data collection has also become a major initiative within the FDA and the PDCs. However, there is a great deal of emphasis on utilizing large data registries to aid in the process. This includes retrospective data acquisition for trial planning and registry and safety data utilization for label expansion. One of the major FDA programs focusing on this endeavor, the National Evaluation System for health Technologies Coordinating Center (NESTcc), was created out of an agreement between the FDA’s CDRH and one of the largest public–private partnerships, the Medical Device Innovation Consortium. By integrating real-world evidence from diverse sources, such as electronic health records, medical registries, claims data, and patient-generated information, NESTcc builds an infrastructure to monitor device performance across various patient populations and care settings. Additionally, NESTcc fosters collaboration among industry, healthcare providers, regulatory agencies, and patient groups, improving transparency and trust in device data. Ultimately, NESTcc aims to make regulatory processes more efficient, enhance patient safety, and drive innovation in medical technology, especially in areas with limited existing data [115]. One of the major reasons for the building of resources and infrastructure is the current lack of available off-label use cases. One example is the utilization of cryoablation nerve block therapy in the adolescent population (12–20 years old) with an adult device that was indicated for use in atrial fibrillation and left atrial appendage management. This was one of the few use cases where the expansion of labeling into the pediatric population was completed. 

## 5. Limitations

Despite the aforementioned advancements in neuromodulation and resources for pediatric device development, there continue to be substantial opportunities for improvement. Because of the mentioned gaps, there are limited case studies demonstrating the logistics and financial incentives for device expansion. Based on the increasing emphasis on RWD and the challenges of randomized controlled trials in the pediatric setting, we would expect continued evolution of use cases over the years. As mentioned, there is a lack of long-term outcomes regarding device-related impact on safety and efficacy, an area of research and interest that we expect will continue to improve. This will also be noted as device resources improve in the pediatric space and more devices are commercialized. Because of the scarcity of data surrounding these subjects, major conclusions cannot be drawn.

## 6. Conclusions

Despite progress in addressing the regulatory and developmental challenges of pediatric neuromodulation devices, significant barriers remain in the pathway from concept to FDA approval. Current efforts, including public–private partnerships, targeted funding mechanisms, and specialized regulatory pathways like the Humanitarian Device Exemption, have mitigated some obstacles. However, the high costs, limited market potential, and lack of pediatric-specific infrastructure continue to limit the widespread development and availability of these devices. Policy adjustments to expand funding opportunities, streamline regulatory processes, and introduce pediatric-focused incentives could bridge this gap. These changes would accelerate innovation, allowing more pediatric patients to benefit from neuromodulation therapies that have shown promise in adult populations, and ultimately fostering a more inclusive and effective healthcare landscape for children with neurological conditions.

## Figures and Tables

**Table 1:** List of approved neuromodulation devices, their indications, and whether they have been FDA-approved for pediatric use.

Device Category	Year FDA-Approved	Pediatric Approval	FDA Indications	Pediatric Off-Label Use	Youngest Age of FDA Approval
Deep brain stimulator (DBS)	1997	✔	Parkinson’s disease, essential tremor, dystonia, OCD, epilepsy	✔	N/A
Vagus nerve stimulator (VNS)	1997	✔	Epilepsy, depression	✔	4 years
Spinal cord stimulator (SCS)	1984		Chronic pain	✔	N/A
Sacral neuromodulation (SNM)	1997		Overactive bladder, urge urinary incontinence, chronic fecal incontinence, non-obstructive urinary retention	✔	N/A
Peripheral nerve stimulation (PNS)	1989		Chronic pain	✔	N/A
Responsive neurostimulation (RNS)	2013		Epilepsy	✔	N/A
Implantable tibial nerve stimulator (iTNS)	2022		Overactive bladder		N/A

## Data Availability

No new data were created or analyzed in this study. Data sharing is not applicable to this article.
